# Health-related quality of life and self-reported health status in adolescents with chronic health conditions before transfer of care to adult health care: an international cohort study

**DOI:** 10.1186/s12887-024-04629-x

**Published:** 2024-03-08

**Authors:** Mira Kallio, Anna Tornivuori, Päivi J. Miettinen, Kaija-Leena Kolho, Heikki Relas, Evelyn Culnane, Hayley Loftus, Susan M. Sawyer, Silja Kosola

**Affiliations:** 1 Department of Pediatrics, Helsinki University Hospital and University of Helsinki, Helsinki, Finland; 2 Department of Nursing Science, University of Turku, Turku, Finland; 3 Pediatric Research Center, Helsinki University Hospital and University of Helsinki, Helsinki, Finland; 4 Faculty of Medicine and Health Technology, Tampere University, Tampere, Finland; 5 Department of Rheumatology, Helsinki University Hospital and University of Helsinki, Helsinki, Finland; 6 Transition Support Service, The Royal Children’s Hospital, Melbourne, Australia; 7 Centre for Adolescent Health, Royal Children’s Hospital, Melbourne, Australia; 8 Murdoch Children’s Research Institute, Melbourne, Australia; 9 Department of Paediatrics, The University of Melbourne, Melbourne, Australia; 10 Research, Development and Innovations, Western Uusimaa Wellbeing Services County, Espoo, Finland

**Keywords:** Adolescents, Chronic medical conditions, Transition, Transfer of care, Health-related quality of life, PedsQL, 16D, Self-reported health

## Abstract

**Background:**

Heath-related quality of life (HRQoL) is lower in adolescents with chronic health conditions compared to healthy peers. While there is evidence of some differences according to the underlying condition and gender, differences by measure and country are poorly understood. In this study we focus on the differences in HRQoL in adolescents with various chronic medical conditions in the year before transfer of care to adult health services. We also study the associations of two different HRQoL measurements to each other and to self-reported health.

**Methods:**

We recruited 538 adolescents from New Children`s Hospital, Helsinki, Finland, and the Royal Children`s Hospital, Melbourne, Australia in 2017–2020. We used two validated HRQoL measurement instruments, Pediatric Quality of Life Inventory (PedsQL) and 16D, and a visual analog scale (VAS) for self-reported health status.

**Results:**

In total, 512 adolescents (50.4% female, mean age 17.8 [SD 1.2] years), completed the survey measures. Higher HRQoL was reported in males than females in both countries (PedsQL 79.4 vs. 74.1; 16D 0.888 vs. 0.846), and in adolescents from Finland than Australia (80.6 vs. 72.2 and 0.905 vs. 0.825, *p* < 0.001 for all). Adolescents with diabetes, rheumatological, nephrological conditions and/or organ transplants had higher HRQoL than adolescents with neurological conditions or other disease syndromes (*p* < 0.001). PedsQL and 16D scores showed a strong correlation to each other (Spearman correlation coefficient *r* = 0.81). Using the 7-point VAS (1-7), 52% (248 of 479) considered their health status to be good (6-7) and 10% (48 of 479) rated it poor (1-2). Better self-reported health was associated with higher HRQoL.

**Conclusions:**

The HRQoL of transition aged adolescents varies between genders, diagnostic groups, and countries of residence. The association between self-reported health and HRQoL suggests that brief assessment using the VAS could identify adolescents who may benefit from in-depth HRQoL evaluation.

**Trial registration:**

Trial registration name The Bridge and registration number NCT04631965 (https://clinicaltrials.gov/ct2/show/NCT04631965).

## Background

The transition of care from pediatric to adult health services can be emotionally demanding and medically risky for adolescents with chronic medical conditions [1, 2]. During this time, clinical variables tend to deteriorate, hospitalizations increase, and follow-up rates decrease [3–5]. This is understandable considering that adolescents are expected to increasingly manage their health independently of their parents, in addition to grappling with the usual challenges of adolescence and young adulthood [2, 6]. 

Besides directly affecting physical health and emotional well-being, chronic medical conditions in adolescence can also impact education and employment, peer relationships and intimacy, as well as recreation and hobbies [7, 8]. Adolescents judge the impact of their chronic medical condition on their ability to participate in normative social, education and recreational pursuits rather than on clinical measures that are more typically the focus of medical professionals [9, 10]. In this regard, patient-reported outcomes such as health-related quality of life (HRQoL) are considered essential in evaluating treatment effectiveness [11, 12]. Indeed, a recent Delphi study found that health professionals considered achieving optimal HRQoL to be the most important outcome of the transition to adult health services [13]. 

HRQoL has been previously shown to be lower in children and adolescents with chronic medical conditions than among healthy peers and to vary according to the underlying medical condition [14–19]. Although HRQoL is considered an important indicator of a successful transition of care, studies of HRQoL among transition-aged adolescents remain relatively scarce [20, 21]. Among adolescents who are close to transferring to adult health care, HRQoL could potentially be affected by the proximity of the transfer itself. To utilize HRQoL as an outcome measure, also pre-transfer measurement should be conducted. Furthermore, to the best of our knowledge, there are no studies that compare different measures of HRQoL in transition-aged adolescents with different chronic medical conditions.

In this study, our aim was to examine HRQoL using two validated HRQoL measurement instruments, Pediatric Quality of Life Inventory (PedsQL) and 16D, as well as self-reported health status using a single-item Visual Analogue Scale (VAS) in a cohort of transition aged adolescents from Finland and Australia. We then aimed to compare the results of these HRQoL measurements with each other and with self-reported health status. Our hypothesis was that adolescents with conditions of greater functional impact and requiring more daily self-care would report poorer HRQoL than adolescents with other conditions. We also hypothesized that self-reported health status would correlate with HRQoL.

## Methods

### Ethics

This study is part of an international observational prospective cohort study called The Bridge [22]. It is approved by the Ethics Committee for Women`s and Children`s Health and Psychiatry at the Helsinki University Hospital, Finland (HUS/1547/2017) and the Royal Children’s Hospital Human Research Ethics Committee, Australia (38035).

### Study design

The protocol of this international observational prospective cohort study has been previously published [22]. In the current study, we conducted cross-sectional analyses of data collected 0 to 12 months before transfer of care to adult services.

### Participants

Participants who were expected to transfer to adult health care within 12 months were recruited from two study centers, the New Children`s Hospital in Helsinki, Finland, and the Royal Children’s Hospital in Melbourne, Australia, between September 2017 and August 2020. In total, 306 consecutive adolescents from Finland and 367 adolescents from Australia were invited to participate. A total of 279 (91%) and 259 (71%) adolescents from each country, respectively, gave written informed consent, and 512 (95% of those who gave informed consent) completed the survey. Participants received compensation valued at 10 EUR/AUD for each completed survey. Eligibility criteria are reported in the Bridge study protocol [22]. 

Participants were categorized into subgroups according to their medical condition. The diagnoses were identified from electronic medical records, and adolescents were categorized into subgroups based on their major diagnosis (endocrinology, gastroenterology, rheumatology, nephrology and/or organ transplants, neurology, cardiology, and others). ‘Others’ included participants from Australia whose condition did not fit the subgroups mentioned above. Most of the adolescents in this group had lung diseases, e.g., cystic fibrose or asthma (n = 16), a syndrome/metabolic condition affecting multiple organs (n = 12), psychiatric conditions, e.g., eating disorders (n = 6), or skin diseases, e.g., epidermolysis bullosa (n = 6). Sixteen adolescents had less common conditions, e.g. immunodeficiency.

### Demographic data

Demographic data included gender and primary language spoken at home.

### Pediatric quality of life inventory (PedsQL)

PedsQL is a self-reported, validated instrument developed to measure HRQoL in children and adolescents aged 8–18 years with chronic or acute health conditions and in healthy populations [18, 23]. The generic score scale has 23 questions in four subgroups (physical, emotional, social, and educational functioning), and each question is answered using a 5-point Likert scale (0 = never a problem and 4 = almost always a problem). Participants are asked to reflect on the last month when answering questions. Items are reverse scored on a 0 to 100 scale, where higher scores indicate better HRQoL. Total Scale scores, Physical Health scores and Psychosocial Health Summary scores (including mean scores for emotional, social, and school functioning domains) are calculated as means of subgroup items. While there are no clinically meaningful cut-off scores, Huang et al. proposed the following cut-off scores for children and adolescents aged 8 to 18 years for total PedsQL scores: minor chronic condition 78 and major chronic condition 70 [24]. 

### 16D

15D is a self-reported, validated measure of HRQoL for adults that has been shown to be reliable and sensitive [25, 26]. Based on the 15D, the 16D was developed for adolescents aged 12–15 years, but has been used up to the age of 18 [27]. 16D measures HRQoL in 16 dimensions: mobility, vision, hearing, breathing, sleeping, eating, speech, excretion, discomfort and symptoms, depression, distress, mental function, vitality, physical appearance, usual activities (school and hobbies) and friends. Participants are asked to reflect on their current situation. Each question has five response options (1 = the best possible situation and 5 = the worst situation). 16D can be used to report a profile across the 16 dimensions or a calculated single index score. The formula for the index score includes importance weights for each dimension, which are combined with participant responses. The optimal index score is 1 and the worst possible score is 0 [28]. 16D has been used to evaluate HRQoL in patients with various diagnoses [16, 29–31]. The minimum important difference is ± 0.015 [32]. The index score can also be utilized to estimate quality-adjusted life years [33]. 

### Self-reported health status

Adolescents estimated their current health status using a single item [34]. In Finland, we asked adolescents to estimate the activity of their condition or the severity of symptoms during the past week. Due to technical limitations with the electronic questionnaire, VAS had to be transformed into a scale from one (very difficult situation) to seven (very good situation). In Australia, adolescents responded to the question “How much has your condition impacted on you during the last week” by using a VAS from zero to a hundred (i.e., not at all to extreme amount). After data collection, the Australian responses were reversed and divided into seven groups to correspond to the Finnish scale. Finally, all responses were divided into three categories: good (scores 6 to 7), moderate (3 to 5) and poor (1 to 2).

### Statistical analysis

We used means with standard deviations (SD) and medians with ranges for continuous variables. To enable comparisons to other HRQoL studies, we mainly used means and SD to describe variables of HRQoL although PedsQL and 16D were not normally distributed (scores were weighed towards the better end). We presented categorical data as frequencies and percentages (%) and ordinal data as medians with interquartile ranges.

We used the Mann-Whitney U test to compare HRQoL between genders and study sites. To study the associations between categorial variables with more than two groups (diagnostic groups and self-reported health) and continuous variables of PedsQL and 16D, we used the Kruskal-Wallis test. The Chi-square test for independence was used to compare categorial variables. We also conducted standard linear multiple regression analyses with PedsQL total scores and 16D single index scores as the dependent variables.

We used the Spearman correlation coefficient to examine correlations between two continuous variables, and the strength of the correlation was classified as very high (0.90 to 1.00), high (0.70 to 0.90), moderate (0.50 to 0.70), low (0.30 to 0.50), and negligible (0.00 to 0.30) [35]. 

Statistical analyses were performed using IBM SPSS Statistics 25 (IBM, Somers, NY). All tests were two-tailed, and *p* < 0.05 was considered statistically significant.

## Results

In total, 512 adolescents (253 from Finland and 259 from Australia) completed the survey with an even balance by gender (Table 1). The mean age of participants was 17.8 (SD 1.2) years (range 15.3–22.8) and the mean age at diagnosis was 7.3 (SD 5.7) years (range 0-17.5). Finnish adolescents were older at diagnosis (mean ages 9.0 and 5.4) and younger at the time of HRQoL assessment (17.2 and 18.4 years, *p* < 0.001 for both). The most common conditions were diabetes (*n* = 151, 29.5%), neurological conditions (*n* = 80, 15.6%) and gastrointestinal diseases (*n* = 72, 14.1%).

**Table 1:** Demographic and clinical characteristics of 512 adolescents with chronic medical conditions

	Total	Finnish	Australian
**Number of adolescents (%)**	**512**	**253 (49.4)**	**259 (50.6)**
Male	250 (48.8)	118 (46.6)	132 (51.0)
Female	258 (50.4)	132 (52.2)	126 (48.6)
Other/missing	4 (0.8)	3 (1.2)	1 (0.4)
**Diagnostic group, n (%)**	**512**	**253**	**259**
Diabetes	151 (29.5)	92 (36.4)	59 (22.8)
Neurology	80 (15.6)	18 (7.1)	62 (23.9)
Gastrointestinal disease	72 (14.1)	45 (17.8)	27 (10.4)
Rheumatology	70 (13.7)	66 (26.1)	4 (1.5)
Other	56 (10.9)	0 (0)	56 (21.6)
Cardiovascular	43 (8.4)	19 (7.5)	24 (9.3)
Nephrology and/or organ transplant	40 (7.8)	13 (5.1)	27 (10.4)
**Mean age (SD)**			
At diagnosis (*n* = 481*) Diabetes Neurology Gastrointestinal disease Rheumatology	**7.3 (5.7**) 7.9 (4.3) 4.3 (5.3) 11.1 (5.0) 10.3 (4.9)	**9.0 (5.3)** 8.1 (4.6) 4.7 (6.0) 11.9 (4.0) 10.7 (4.7)	**5.4 (5.6)** 7.4 (3.7) 4.1 (5.2) 9.8 (6.1) 4.8 (5.6)
Other Cardiovascular Nephrology and/or organ transplant **At survey completion (*n* = 511)**	4.3 (5.8) 5.2 (6.6) 4.2 (5.3) **17.8 (1.2)**	- 6.3 (6.8) 6.6 (6.3) **17.2 (1.2)**	4.3 (5.8) 4.3 (6.5) 2.7 (4.1) **18.4 (0.7)**
**Language spoken at home, n (%)**	*n* = 489	*n* = 253	*n* = 236
English	233 (47.6)	0 (0)	233 (98.7)
Finnish	210 (42.9)	210 (83.0)	0 (0)
Finnish and another language	27 (5.5)	27 (10.7)	0 (0)
Other languages	19 (3.8)	16 (6.3)	3 (1.3)

* Data from 31 Australian adolescents were not available

There was no significant difference between genders between study sites (*p* = 0.42). The number of adolescents in different diagnostic groups was significantly different between study sites (*p* < 0.001). Adolescents from Finland were older at time of diagnosis (*p* < 0.001) and younger at the time of completing the survey than adolescents from Australia (*p* < 0.001)

### HRQoL and self-reported health status

Of the 512 participants, 95% completed the PedsQL and 16D (Table 2). The mean total PedsQL score was 76.5 (SD 16.2). HRQoL scores were highest in social functioning 83.8 (SD 18.1) and lowest in school functioning 69.5 (SD 19.4). The 16D mean single index score was 0.87 (SD 0.10). The median score for the VAS (*n* = 479) was 6 (good), while 52% (*n* = 248) rated their situation as good and 10% (*n* = 48) as poor.

**Table 2:** Self-reported health status (medians) and HRQoL (means)

	All *n* = 479 to 493**	FIN *n* = 251 to 252	AUS *n* = 235 to 242	Males *n* = 225 to 239	Females *n* = 245 to 250
**VAS**					
** Score (1–7)** ** IQR**	**6.0** 4–7	**6.0** 4–7	**6.0** 3–7	**6.0** 4–7	**5.0** 4–7
**PedsQL**					
** Total (SD)** Physical functioning Psychosocial functioning Emotional functioning Social functioning School functioning	**76.5 (16.2)** 79.5 74.9 71.2 83.8 69.5	**80.6 (13.1)** 85.1 78.1 74.1 88.0 72.3	**72.2 ª (18.1)** 73.4 ª 71.4 ª 68.0 ^b^ 79.4 ª 66.6 ^b^	**79.2 (15.5)** 82.1 77.5 75.9 84.5 72.0	**74.1** ^**c**^ **(16.6)** 76.9 ^c^ 72.6 ^d^ 67.1 ^c^ 83.4 67.3 ^d^
**16D**					
** Total (SD)** Vitality Vision Breathing Distress Hearing Sleeping Eating Discomfort and symptoms Speech Physical appearance School and hobbies Mobility Friends Mental function Excretion Depression	**0.866 (0.10)** 0.779 0.927 0.850 0.717 0.969 0.762 0.981 0.795 0.928 0.774 0.814 0.972 0.877 0.918 0.896 0.806	**0.905 (0.08)** 0.811 0.935 0.941 0.792 0.976 0.816 0.995 0.827 0.951 0.862 0.855 0.989 0.949 0.947 0.911 0.832	**0.825**^**a**^ * (0.11) 0.745 ^b^ * 0.919 * 0.756 ª * 0.639 ª * 0.961 * 0.706 ª * 0.967 ^b^ * 0.761 ^b^ * 0.903 ª * 0.683 ª * 0.771 ª * 0.953 ª * 0.801 ª * 0.888 ª * 0.880 * 0.778 ^b^ *	**0.888 (0.10)** 0.834 0.947 0.869 0.779 0.974 0.785 0.977 0.835 0.925 0.839 0.846 0.968 0.886 0.929 0.907 0.841	**0.846** ^**c**^ * (0.10) 0.728 ^c^ * 0.908 ^c^ * 0.833 * 0.661 ^c^ * 0.963 0.743 * 0.985 0.756 ^c^ * 0.932 0.714 ^c^ * 0.784 ^d^ * 0.975 0.870 * 0.908 * 0.883 * 0.776 ^d^ *

HRQoL = health-related quality of life, FIN = adolescents from Finland, AUS = adolescents from Australia. VAS = Visual Analogue Scale, IQR = interquartile range, SD = standard deviation. a = difference between countries is significant at *p* < 0.001 level (Mann-Whitney U test). b = difference between countries is significant at *p* < 0.05 level (Mann-Whitney U test). c = difference between genders is significant at *p* < 0.001 level (Mann-Whitney U test). d = difference between genders is significant at *p* < 0.05 level (Mann-Whitney U test). *= difference between countries or genders in 16D is more than the minimum important change (± 0.015). ** = the number of adolescents who completed VAS, PedsQL and 16D varied

There was no significant association of age at diagnosis and age at survey assessment with PedsQL total scores or 16D single index scores (*r*= -0.26 to 0.11) according to Spearman correlation coefficients. However, in multiple regression analysis concerning 16D single index scores, younger age was associated with better scores (*p* = 0.018, Table 3).

**Table 3:** Results of standard multiple regression. **(A)** with PedsQL total scores as the dependent variable. **(B)** with 16D single index scores as the dependent variable

	Unstandardized β Coefficient	Standardized β Coefficient	*P*-value	95% Cl for unstandardized β	
A				Lower	Upper
Gender *	-5.307	-0.165	**<0.001**	-8.109	-2.505
Country **	-7.092	-0.220	**<0.001**	-10.463	-3.722
Age at diagnosis	0.153	0.055	0.242	-0.103	0.409
Age at survey completion	-0.919	-0.068	0.183	-2.274	0.436
	**Unstandardized β** **Coefficient**	**Standardized β** **Coefficient**	***P-value***	**95% Cl** **for unstandardized β**	
**B**				**Lower**	**Upper**
Gender *	-0.044	-0.213	**<0.001**	-0.061	-0.027
Country **	-0.074	-0.353	**<0.001**	-0.094	-0.053
Age at diagnosis	-0.001	-0.043	0.323	-0.001	0.001
Age at survey completion	-0.010	-0.114	**0.018**	-0.018	-0.002

Gender as 1 = male and 2 = female. ** Country as 1 = Finland and 2 = Australia. *P* < 0.05 is considered as statistically significant

### Differences between genders

**Fig. 1:**
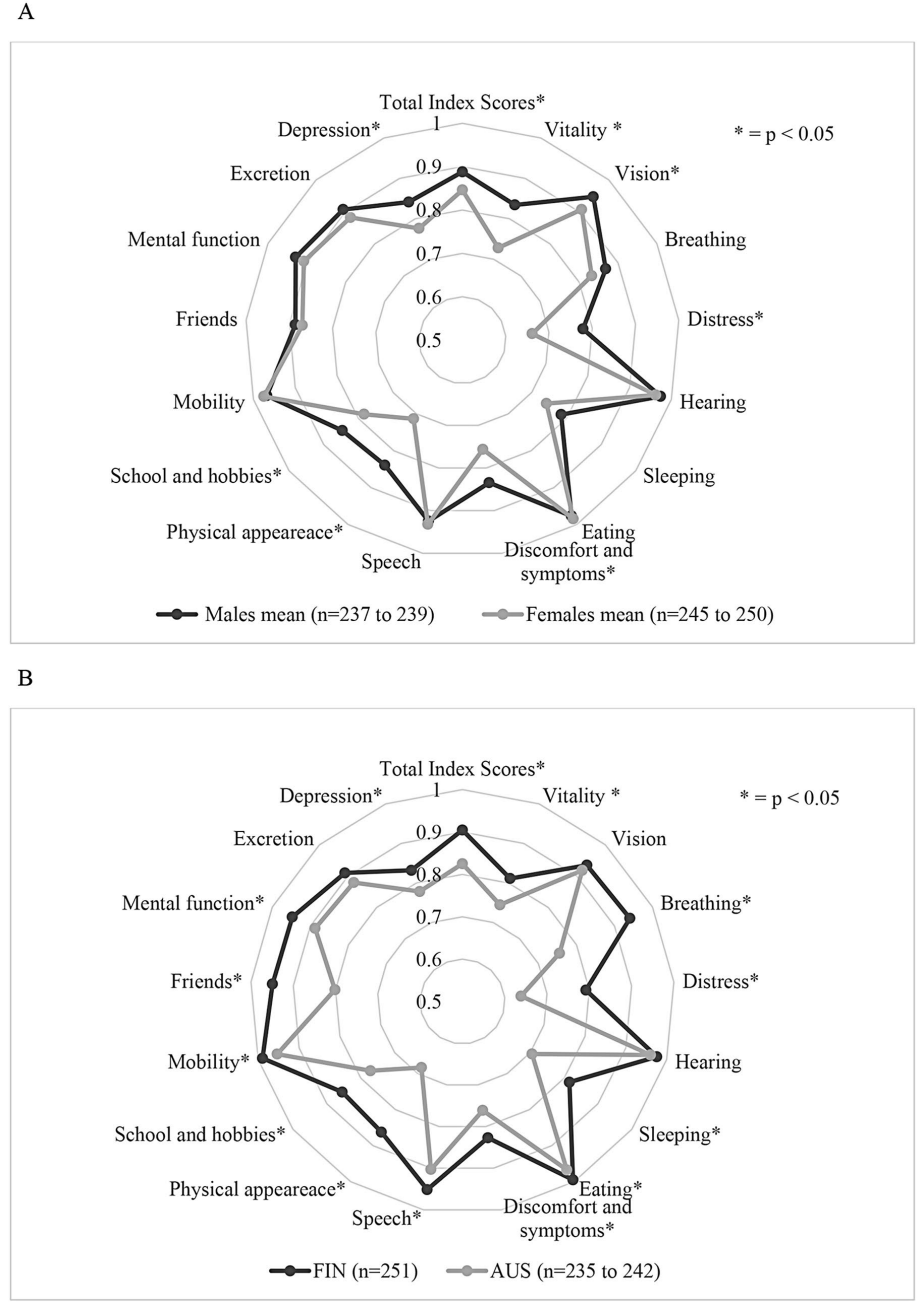
Differences in 16D by gender (**A**) and study site (**B**). (**A**) 16D mean scores by gender (male, female). (**B**) 16D mean scores by country (FIN = Finland, AUS = Australia)

Males had higher PedsQL total scores (mean score 79.2 [SD 15.5]) than females (mean score 74.1 [SD 16.6]; *p* < 0.001) (Tables 2 and 3). The difference in scores was greatest in emotional functioning (75.9 vs. 67.1; *p* < 0.001) (Table 2). The same gap in scores between genders was found at both study sites. Males also had higher overall scores on the 16D (mean scores 0.89 vs. 0.85 [SD 0.1 for both]; *p* < 0.001) (Tables 2 and 3; Fig. 1). On the 16D, females reported lower HRQoL scores than males in a variety of dimensions, including vitality, vision, distress, discomfort and symptoms, physical appearance, school and hobbies, and depression (*p* < 0.05 for all). VAS scores were similar between genders (male median score 6.0 vs. female median score 5.0; *p* = 0.33).

### Differences between study sites

Finnish adolescents reported higher PedsQL and 16D total scores (*p* < 0.001) than Australian adolescents (Tables 2 and 3). Finnish adolescents scored higher in all PedsQL subdomains (*p* < 0.05) and in all 16D dimensions (Table 2; Fig. 1). Self-reported health status was similar by country (*p* = 0.12) (Table 2).

### Differences between diagnostic groups

**Fig. 2:**
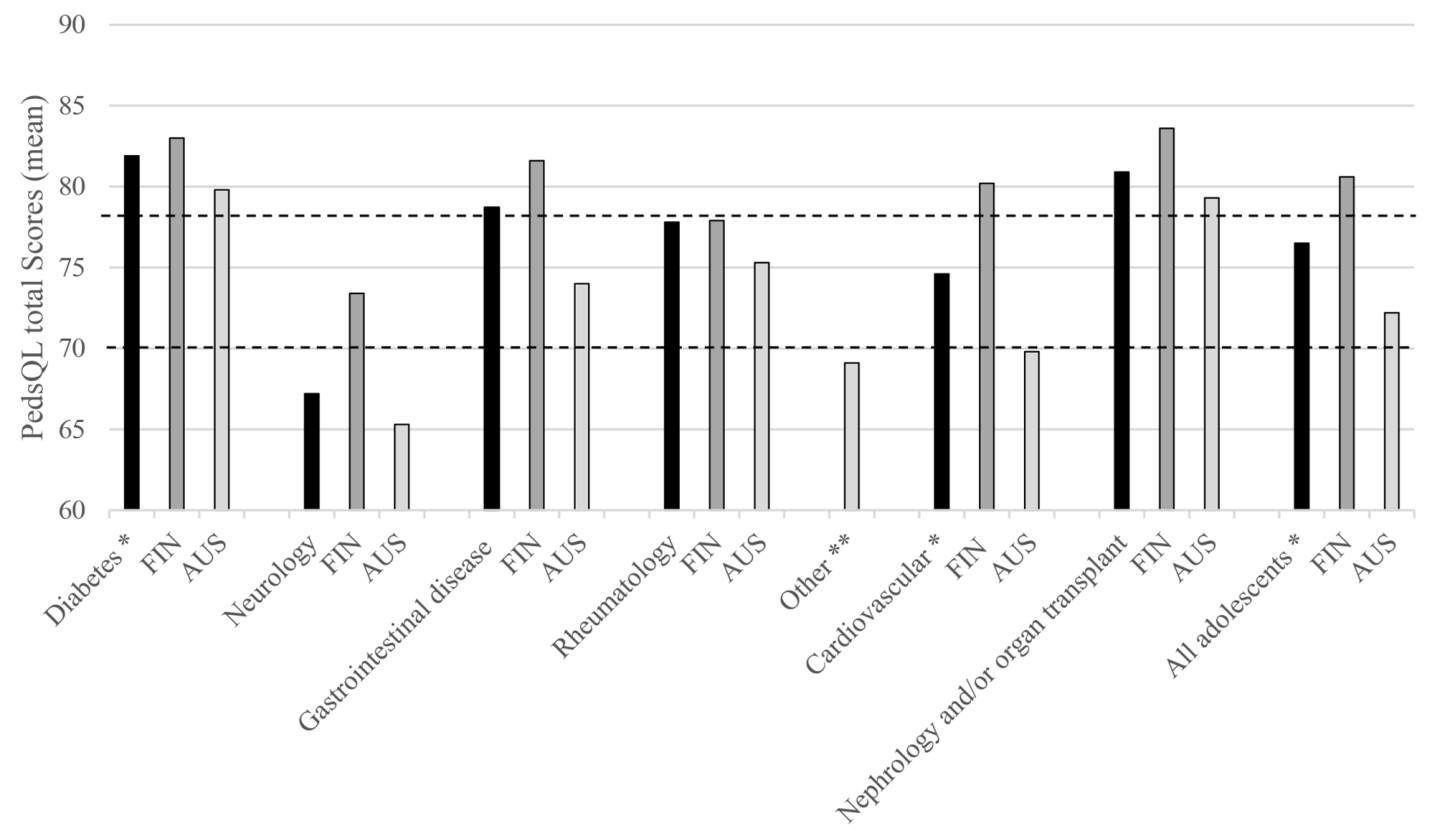
Mean total PedsQL scores by country and diagnostic group. * Difference between countries is statistically significant; *p* < 0.05. ** Diagnostic group other includes adolescents from Australia whose condition did not fit the other diagnostic groups (for example cystic fibrose, eating disorders, and syndromes affecting multiple organs)

**Fig. 3:**
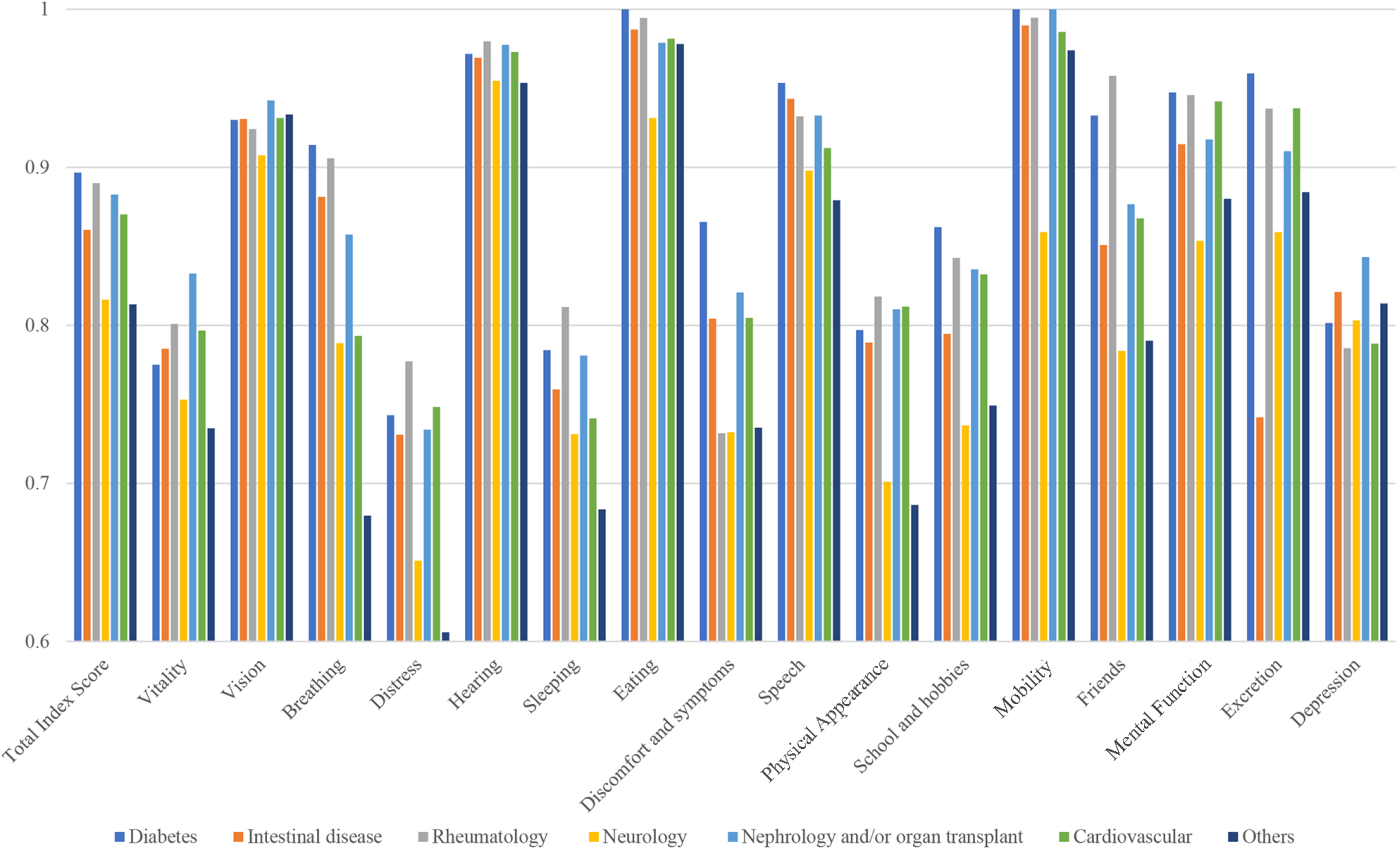
16D mean scores of adolescents according to different diagnostic groups

Adolescents with diabetes and nephrological disease and/or organ transplants had the highest mean total PedsQL scores, while adolescents with neurological conditions and the “other” diagnostic group had the lowest total scores (*p* < 0.001 between diagnostic groups). In both countries, adolescents with diabetes had the highest HRQoL scores (Fig. 2). Adolescents with neurological conditions or in the “other” diagnostic group reported the lowest 16D scores (*p* < 0.001 between groups) (Fig. 3).

Dashed lines are the cut off points suggested by Huang et al. (i.e. 78 for minor chronic condition and 70 for major chronic conditions). Ref [24]. 

### Correlations between HRQoL measurements and HRQoL and self-reported health

**Fig. 4:**
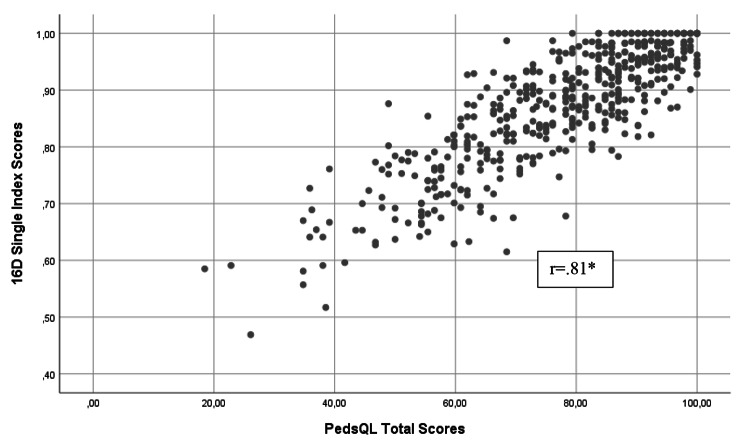
Correlation between PedsQL Total Scores and 16D Single Index Scores. * Spearman correlation coefficient

The two HRQoL measurements showed strong correlation with each other (Spearman correlation coefficient *r* = 0.81) (Fig. 4). The correlation remained strong in subgroup analyses including gender, study sites and diagnosis groups (data not shown).

**Fig. 5:**
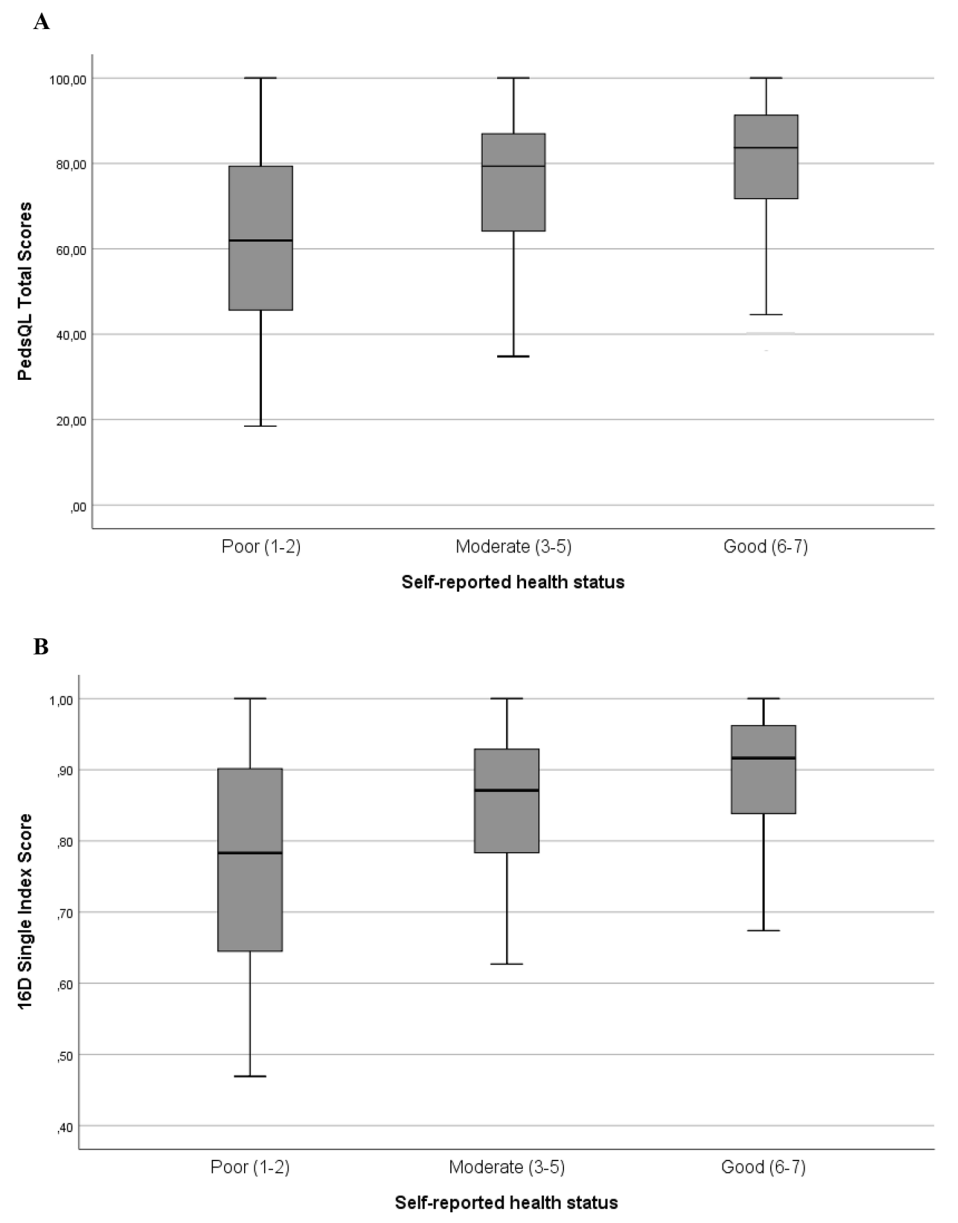
Self-reported health status and health related quality of life. **(A)** PedsQL Total Scores and Self-reported health status. **(B)** 16D Single Index Scores and Self-reported health status

Better self-reported health was associated with better HRQoL scores. Mean (median) PedsQL total scores of adolescents who estimated their health status as good, moderate, and poor on the VAS were 80.6 (83.7), 75.1 (79.4) and 62.9 (62.0) and the mean (median) 16D index scores were 0.90 (0.92), 0.86 (0.87) and 0.77 (0.78), respectively (Fig. 5).

## Discussion

In this study of adolescents with chronic health conditions prior to transfer to adult healthcare, males had higher HRQoL than females, and adolescents from Finland had higher HRQoL than adolescents from Australia. The two HRQoL measurements, PedsQL and 16D, correlated strongly with each other, with 16D providing more nuanced information between diagnosis groups. Both HRQoL measures were closely associated with self-reported health status.

The HRQoL of adolescents in our study was lower compared to the HRQoL of healthy adolescents in previous studies and comparable to previous studies conducted in children and adolescents with chronic conditions [24, 36, 37]. This was true in studies which included various conditions and studies focusing separately on diabetes, juvenile idiopathic arthritis, and inflammatory bowel disease [15, 16, 18, 37, 38]. 

We found no significant association between HRQoL measurements and duration of the chronic condition. In multiple regression, the age at HRQoL assessment showed no association with PedsQL total scores and a modest association with 16D single index scores. Previous studies have also yielded mixed results on the association of age and HRQoL [39, 40]. In our study, adolescents completed the survey close to their transfer to adult health care. This upcoming change may have caused distress and thus affected HRQoL. Causal effects are, however, impossible to estimate based on our cross-sectional research.

In our study, males had higher PedsQL and 16D scores than females, consistent with studies conducted in the general population and among adolescents and young adults with chronic conditions [38, 39, 41–44]. Using the 16D, the difference between genders was especially wide in dimensions of vitality, distress, discomfort and symptoms, and physical appearance which may reflect the higher prevalence of mental health symptoms among girls [45, 46]. Females may worry more about their condition and have higher demands toward themselves which may also impact HRQoL [47]. In previous studies of healthy adolescents, the female puberty process and menstrual health have been proposed as one explanatory factor for the gender differences in HRQoL [48]. Since many chronic conditions may affect puberty and menstrual health, these phenomena could act as contributing factors also in our study.

Interestingly, the Finnish cohort reported higher HRQoL than the Australian cohort. This may in part be explained by differences in the distribution of adolescents across the various diagnostic groups, as more Australian participants had neurological and “other” conditions which, as a group, reported lower scores. However, Australian adolescents estimated their HRQoL to be lower across all diagnostic subgroups (e.g., diabetes). While Australian adolescents were older when completing the survey and younger at diagnosis, we found no significant association between age and HRQoL. Other possible explanations might be cultural differences related to education, socioeconomic status, national happiness, or differences in experiencing hardships [49–51]. 

Adolescents with diabetes reported better HRQoL than adolescents with rheumatologic or neuromuscular disorders. While consistent with other studies [19, 52] it is slightly surprising how little the intense self-management requirements in diabetes were reflected in lower HRQoL. Factors that impact HRQoL of adolescents with chronic conditions around transfer of care are only partly understood. In previous studies, factors associated with inferior HRQoL during transition included female gender, high disease activity and pain, psychiatric comorbidity, smoking, and obesity [20, 38, 53]. Satisfaction with care and transition readiness may be positively connected to HRQoL among adolescents and young adults, but the results are inconclusive [52, 54–57]. 

Despite different time frames of focus within the two measures of HRQoL, the findings were strongly correlated (*r* = 0.81), affirming their suitability for this cohort. Notwithstanding wide use of both measures, we found only two previous studies that directly compared the 16D and PedsQL. Mört et al. reported a moderate correlation between these HRQoL instruments (*r* = 0.40–0.65) in a population of cancer survivors (*n* = 203, age 11–18 years) and their controls, and Kyösti et al. found commensurate HRQoL in children and adolescents discharged from the intensive care unit (*n* = 1109) [29, 58]. The 16D provides more detail on physical wellbeing due to specific questions regarding breathing, vision, hearing, eating, speaking, physical appearance and excretion compared to the focus on walking, running and physical exercise in the PedsQL. When comparing HRQoL of patients with different chronic conditions, the 16D may thus be more sensitive to underlying reasons for poorer HRQoL. While specific impacts of different conditions may render a decision to use one or the other more logical, both measures addressed the more generic domains of sleep and schooling which were found to be so important.

In this study, self-reported health status correlated with HRQoL, and better health scores from this single item indicated better HRQoL. However, there was significant overlap in HRQoL scores across self-reported health categories so poor self-reported health does not necessarily mean poor HRQoL. In previous studies, symptoms affecting daily life (evaluated as VAS 1–7) and lower HRQoL reflected higher disease activity (estimated by professionals) among children and adolescents with inflammatory bowel disease, and the VAS was especially useful to recognize patients who need psychosocial support [16, 34]. Self-rated health measured by a 0-100 VAS also correlated with PedsQL total scores in a study of adolescents with congenital heart disease [59]. In clinical use, self-reported health status could facilitate recognition of adolescents who could benefit from more support. This evaluation could be supplemented with in-depth HRQoL assessments, as recommended in adolescents with diabetes based on the finding that lower HbA1C was strongly associated with better HRQoL in an international study of young people with type 1 diabetes [39]. While the PedsQL or 16D can also serve as openers for more detailed discussions of personal life difficulties caused by the medical condition [60], they are longer measures and more challenging to incorporate into routine care. Considering the benefits and disadvantages of different instruments, we suggest routine use of self-reported health evaluation when assessing the effects of chronic conditions on adolescent lives and deepening the discussion of different areas of life as needed.

Future research is needed for more in-depth evaluation of the reasons behind the differences in HRQoL in different populations. Also, prospective studies measuring self-reported health and HRQoL would give more information about the responsiveness to change of these measurements during the transition process.

The strengths of this study include the international setting, the range of medical conditions, and high participation rates at both study sites. To the best of our knowledge, this study is the first to conduct international comparisons of HRQoL in adolescents with multiple chronic medical conditions preparing for transfer to adult health services. We used two generic, self-reported HRQoL instruments and found similar results. One limitation of our study is that no unambiguously ‘normal’ values exist for either the PedsQL or the 16D, and we had no reference group of healthy adolescents in either country. Both the question wording and response options for the VAS question varied slightly in the Australian and Finnish surveys, but the scores were very similar. While it was straightforward to convert these scores for statistical analysis, we do not know if there are any intrinsic differences in how adolescents complete a score from 1 to 7 versus 0-100. The study population consists of adolescents close to transfer of care in two industrialized countries, and the results could be very different among populations of different ages or adolescents in developing countries.

## Conclusions

In this cohort of transition aged adolescents from Finland and Australia, HRQoL varied between medical conditions, genders, and country. Given its correlation with HRQoL, the use of a single self-reported question of health status may have utility as a screening tool among adolescents with chronic medical conditions to identify adolescents who may need more support.

## Data Availability

The datasets used during the current study are available from the corresponding author on reasonable request.

## Abbreviations

AUS: Adolescents from Australia
FIN: Adolescents from Finland
HRQoL: Health related quality of life
IQR: Interquartile range
PedsQL: Pediatric Quality of Life Inventory
SD: Standard deviation
VAS: Visual Analogue Scale
